# Historical contingency impacts on community assembly and ecosystem function in chemosynthetic marine ecosystems

**DOI:** 10.1038/s41598-021-92613-1

**Published:** 2021-07-07

**Authors:** Dimitri Kalenitchenko, Erwan Peru, Pierre E. Galand

**Affiliations:** 1 CAGE - Centre for Arctic Gas Hydrate, Environment and Climate, Department of Geosciences, UiT The Arctic University of Norway, Tromsø, Norway; 2 Sorbonne Université, CNRS, Laboratoire d’Ecogéochimie des Environnements Benthiques (LECOB), Observatoire Océanologique de Banyuls, 66500 Banyuls-sur-Mer, France

**Keywords:** Microbial ecology, Microbial biooceanography

## Abstract

Predicting ecosystem functioning requires an understanding of the mechanisms that drive microbial community assembly. Many studies have explored microbial diversity extensively and environmental factors are thought to be the principal drivers of community composition. Community assembly is, however, also influenced by past conditions that might affect present-day assemblages. Historical events, called legacy effects or historical contingencies, remain poorly studied in the sea and their impact on the functioning of the communities is not known. We tested the influence, if any, of historical contingencies on contemporary community assembly and functions in a marine ecosystem. To do so, we verified if different inoculum communities colonizing the same substrate led to communities with different compositions. We inoculated wood with sea water microbes from different marine environments that differ in ecological and evolutionary history. Using 16S rRNA and metagenomic sequencing, it was demonstrated that historical contingencies change the composition and potential metabolisms of contemporary communities. The effect of historical events was transient, dominated by environmental selection as, over time, species sorting was a more important driver of community assembly. Our study shows not only that historical contingencies affect marine ecosystems but takes the analysis a step further by characterizing this effect as strong but transient.

## Introduction

Many ecosystem processes on Earth are driven by microbes. The composition and diversity of microbial communities thus have a strong and direct influence on ecosystem function^1–5^. A good predictor of microbial ecosystem function requires an understanding of the mechanisms that drive community assembly. Patterns of community assembly can be shaped by stochastic/neutral processes, but also by biotic interactions, historical contingencies, and the current environmental conditions (abiotic factors)^6^. Environmental conditions, including temperature, number of hours of sunlight per day and nutrient concentrations, are thought to be the principal drivers of archaeal and bacterial community composition globally^7–10^. However, many environmental studies on microbial communities are only snapshots of the patterns of diversity that do not consider the historical processes. Community assembly can indeed be influenced by past biotic and abiotic conditions that might affect present-day assemblages. The effects of past historical events on contemporary communities are called legacy effects or historical contingencies^11^. Such effects may be due to past environmental conditions that shaped the communities, but they may also result from limited dispersal in the past that influences the composition of communities^12^, or they may be priority effects resulting from the order or timing in which different species joined a community^11^.

Studies have been conducted to specifically test the effect of historical contingencies on present-day communities by examining soils exposed to drought and/or precipitations within the context of climate change^13^. Effects of historical contingency on community diversity and composition have been observed in some cases^14,15^, but not all^16^. The history of the communities was shown to have a stronger effect on aquatic ecosystems than environmental conditions^17^ but this could depend on the habitat^18^. Many studies have focused on the priority effect, which can be seen as one of the causes of historical contingencies^19^. The priority effect was identified as important for community assembly in transplant experiments between fresh and brackish water^20^, or between dissimilar ponds^21^. Past environmental conditions have also been considered and have proven to be better predictors of spatial differences in freshwater bacterioplankton communities than contemporary environmental conditions^22^. In addition, dispersion limitation, when considered as a historical contingency, was shown to influence diatom richness in lakes^23^. An experiment conducted on marine ecosystems which manipulated the assembly history of diatoms based on successive inoculations of different strains, found a significant priority effect on community composition^24^. Information on marine ecosystems remains scarce however, and the few studies that exist are based on cultured strains and not on natural communities. Furthermore, they are based on taxonomic information only and do not consider functional genes.

The objective of our study was to test the effect of historical contingencies on community assembly and function in a marine ecosystem. To do so, we examined whether different inoculum communities colonizing the same substrate could lead to communities with different compositions. We hypothesized that the historical contingency would be transient^19^ and that environmental filtering would become the more important driver of assembly over time. To answer this question, an experimental approach that monitors historical contingencies and current environmental conditions was needed^6^. We already showed that wood incubated in seawater is a useful tool for testing hypotheses on the functional ecology of communities^25,26^. Here, wood logs incubated in aquaria were inoculated with sea water microbes from either surface or deep waters. Due to stark and stable environmental dissimilarities between these depths, the inoculation communities have been shaped by different evolutionary and ecological events whose legacies have been maintained. In this experiment, the wood represented a well-defined habitat that was open to colonization by marine species marked by historical contingencies. We monitored the diversity of microbial communities in the wood over time using 16S rRNA sequencing, described their metabolic potential using metagenomic sequencing, and measured the growth of microbial mats on the wood as an indicator of microbial activity.

## Materials and methods

### Experimental set up

Three independent treatments were used in the experiment: (1) wood incubated in a 3 m deep water inoculum (3 m), (2) wood in a 500 m deep water inoculum (500 m) and (3) wood in a 3 m deep water inoculum with continuous renewal of the inoculating water (Open). The 3 m treatment tank (3 m) was initially filled with coastal seawater from the SOLA station in Banyuls Bay sampled from a depth of 3 m on 27th January 2015. The 500 m treatment tank (500 m) was filled with water sampled the same day from a depth of 500 m at the MOLA station (500 m). The microbial community composition of the two sampled waters are significantly different because they are taken from the surface and from deep water masses, which are known to harbor different microbial communities^10,27–29^.

For these two first treatments (3 m and 500 m), wood logs were immersed without water renewal for 9 days. After that, the aquarium water was replaced with bacteria-free water that was continuously renewed until the end of the experiment. To do so, coastal seawater was continuously pumped through a UF100LL filtration module (Polymem, Castanet-Tolosan, France) with a 0.01 µm pore size membrane followed by a 0.2 µm sterivex cartridge (Millipore, MA, USA). The flow rate of the bacteria-free water meant that it was renewed at least twice each day (3.3 L/h). For the open treatment, the aquarium was filled with water taken from a depth of 3 m, but the water was continually renewed from the start of the experiment, using the same coastal seawater which contains the natural microbial communities found in the environment during the experiment (no filtration). In addition to being a positive control, the third treatment tests the priority effect in which new communities are continuously in contact with the wood.

Experiments were conducted in 45-L aquaria (60 × 30 × 25 cm) with each aquarium containing four fresh pine wood logs (10 cm long and 15 cm diameter) cut from the same branch and randomly distributed between the three aquaria^25^. The wood was not autoclaved to avoid altering the integrity of the chemical composition (possible impact on sugars and lipids). However, few bacteria are found naturally in living wood, and a previous experiment showed no rDNA amplification of terrestrial microbes following immersion in seawater, which induces an osmotic shock for the microbial cells^26^. All aquaria were equipped with a diffusor connected to an air compressor to ensure continuous oxygen saturation of the water.

The experiment was conducted for 40 days in the dark with the water temperature maintained at 13 °C, corresponding to the normal temperature of the pumped coastal water. One wood log was removed and sampled from each aquarium after 8 days (8d), 19 days (19d), 28 days (28d) and 40 days (40d). Removing the logs did not significantly change the water level (one wood log represents ca. 4% of the tank volume). At each sampling interval, four identical cores were extracted from each log using a 4.35-mm-wide increment core borer. The four identical cores were taken along a same growth ring 2 cm from the bark. Four replicate cores per sampling interval provide the necessary coverage of the micro variability of the wood^26^. It was preferable to sample four cores from the same removed log instead of drilling one core per immerged wood log to reduce the risk of influencing the wood’s redox gradient. Cores drilled in the logs that remained in the aquaria would have promoted oxygen diffusion inside the wood.

### Image acquisition and measure of microbial mat cover

The white mats growing on the surface of the wood were found to be composed of sulfur-oxidizing bacteria^25^ and their presence thus reflects the production of sulfur in the wood. The microbial mat cover was monitored by image analysis^30^. The aquaria were equipped for the duration of the experiment with a fixed GoPro Hero 3+ camera (GoPro, San Mateo, CA, USA) and 2 LED lights (Aquavie, Connaux, France). An external timer simultaneously started the LED lightning, stopped the air bubbling and triggered the camera every 4 h. A hardware controller (Camdo, Vancouver, BC, Canada) and a custom script in the camera’s SD card allowed the camera to be switched on, take pictures and then be switched off. At the end of the experiment, pictures were converted into 8-bit images using ImageJ software (V.1.52, https://imagej.nih.gov/ij/) and grouped into a stack. For each wood log, a value for the average grey pixel value over the wood surface was calculated. The area covered by the white mats was estimated by measuring changes in grey pixel values. Minimum grey pixel values of time-series images were used to normalize data between logs and aquaria.

### DNA extraction, 16S rRNA and metagenomics sequencing

A 1 cm long sub-sample of the wood core was taken at 4–5 cm depth from inside the wood. The samples were powdered as described earlier^31^. After mixing the wood powder with the extraction buffer from the Maxwell 16 Blood DNA Purification kit, the obtained cell lysate was transferred to the Maxwell 16 Blood DNA Purification kit cartridge and processed automatically with the Maxwell 16 automated extractor (Promega, Fitchburg, MA, USA).

A portion of the 16S rRNA gene was amplified using bacteria specific primers, 28F (5′-TTTGATCNTGGCTCAG-3′) and 519R (5′-GTNTTACNGCGGCKGCTG-3′), and then sequenced by a commercial laboratory (Research and Testing Laboratory, Lubbock, TX) on an Illumina Miseq sequencer to produce 2 × 300 bp long, paired-end sequences. The raw sequence data have been deposited in the NCBI Sequence Read Archive (accession no. SRP099419 and BioProject accession no. PRJNA374511).

For a functional description of the wood communities, eight samples from inoculum at 3 and 500 m depth were selected for shotgun metagenome sequencing as in Kalenitchenko et al.^32^. Because of financial constraints, we could not sequence the open circulation samples for metagenomic analysis. Libraries were prepared with 50 ng of DNA using the Nextera DNA sample preparation Kit (Illumina, San Diego, CA, USA) producing a library with an average insert size of 200 bp. The sequencing was performed on a Hiseq 2 × 150 bp Illumina sequencer by a commercial laboratory (Research and Testing Laboratory).

### Sequence analysis

The 16S rRNA sequences were processed following Galand et al.^33^. In brief, sequences were paired, producing ca. 450 bp fragments, quality trimmed, and chimeras were removed. Sequences were grouped in operational taxonomic units (OTU) at 97% similarity using the Uclust algorithm^34^. The taxonomy of the most abundant sequence of each OTU was assigned using the SILVA SSU 123 database^35^. All samples were resampled down to 5094 sequences per sample, which corresponds to the smallest sample size.

Metagenomic data were processed following Kalenitchenko et al.^32^. Paired-end reads were joined with a minimum overlap setting of 8 bp and a maximum difference of 10%. Both paired and unpaired reads were retained for further analysis. Low-quality regions (phred score ≤ 15) were trimmed using SolexaQA^36^. We chose to analyze unassembled reads because the goal here was to annotate as many reads as possible rather than to reconstruct large bins. A machine-learning approach known as FragGeneScan^37^ identified open reading frames that were annotated using BLASTX against the M5NR^38^, KEGG^39^ and Silva^35^ databases. Sequence counts were normalized with Deseq^40^. Analyses were conducted using the MG-RAST pipeline^41^, and sequences are available under the accession numbers mgs290945, mgs290948, mgs290957, mgs290960, mgs290963, mgs290966, mgs290969, mgs290972.

In order to verify differences in metabolic potential between experimental conditions, we focused particularly on genes known to be present during the degradation of wood in the sea^25,32^. The following genes were targeted: heterodisulfide reductase (hdr) associated with metabolisms of methanogenic archaea; periplasmic nitrate reductase (*napB*); respiratory nitrate reductase (Nar); acetaldehyde/alcohol dehydrogenase gene (*adhE*); ATP citrate lyase (*ACLY*) for anaerobic C fixation; ketoglutarate:ferredoxin oxidoreductase (*korA*) for the anaerobic C fixation in reductive TCA cycle; dissimilatory sulfite reductase (*dsrA*) and adenylylsulfate reductase (*aprA*).

### Statistics

Differences in community diversity were tested using ANOVA with the assumption that each sample from the same log was independent of each other because the distance between samples was ten times higher than the sampled area. The Bray–Curtis dissimilarity index was computed to compare the community composition between samples using an MDS analysis. The effect of inoculate and time factors on composition differences was tested with PERMANOVA in R. A SIMPER test was performed to identify the OTUs that made the largest contribution to the differences between groups. One outlier sample (T4-500m2) was removed from the analysis. The dispersion of the bacterial communities based on the beta diversity was calculated using the vegan package^42^ in R.

## Results

### Microbial mat cover

**Figure 1:**
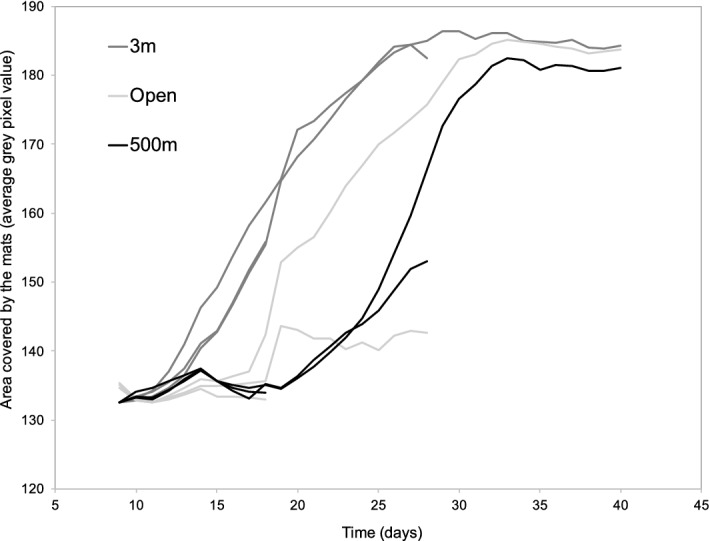
a. Continuous measure of the area covered by the microbial mats growing on the logs in aquaria inoculated with sea water from 3 and 500 m depth, and open circulation. The lines stop when the wood logs were sampled for bacterial community analysis after 19 days, 28 days and 40 days.

We measured the area covered by the microbial mats on the surface of each log using image analysis (Fig. 1). Mats first became visible after 10 days of incubation on those logs inoculated with water from a depth of 3 m depth. Mats then appeared on the logs in the open circulation aquarium, and finally, on the logs incubated with water from a depth of 500 m. For the inoculum from both 3 and 500 m depths, the replicate logs appeared to have similar patterns of mat coverage. For the open circulation logs, one of the logs did not follow the same mat colonization pattern as the other (Fig. 1).

### Bacterial community composition

**Figure 2:**
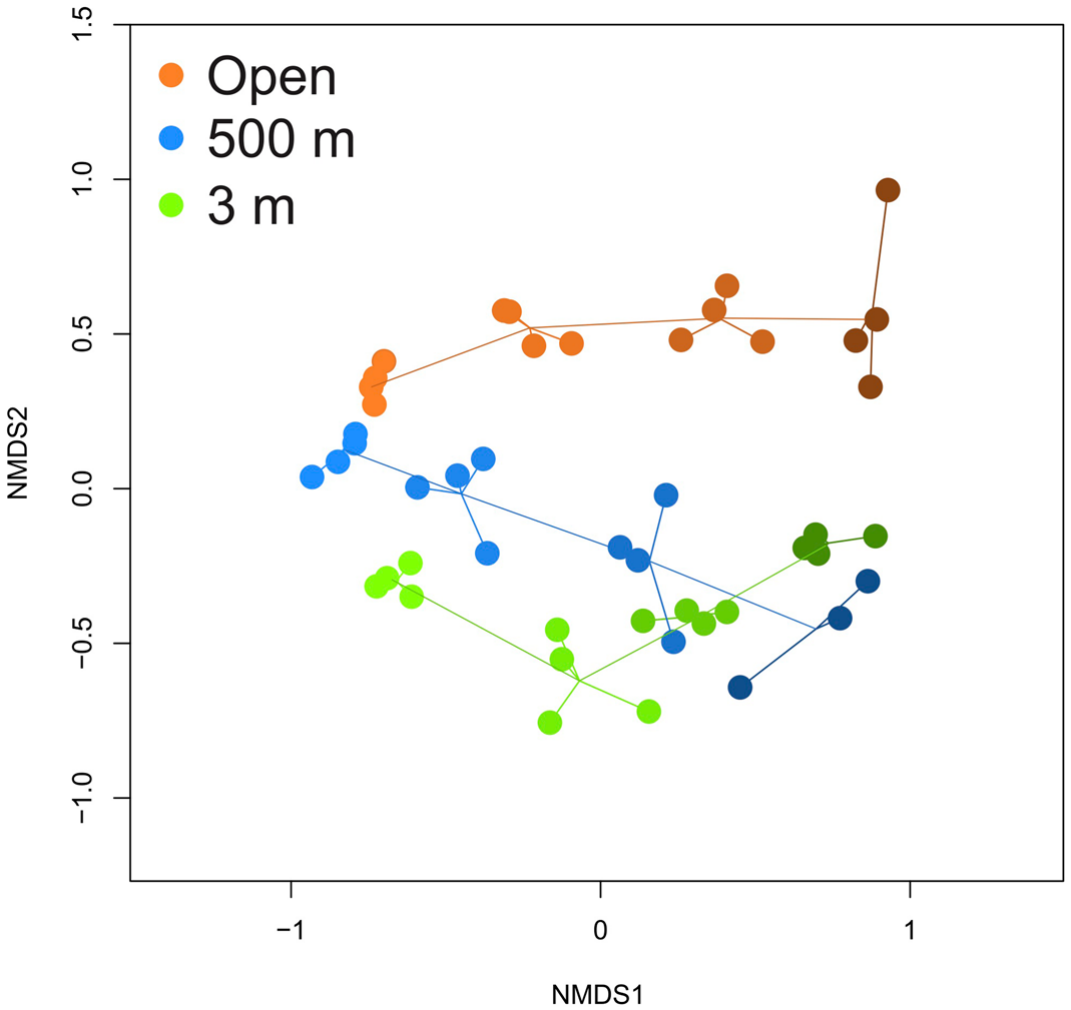
Trajectories of the bacterial communities. The similarity between bacterial communities growing in the wood is represented by an MDS ordination based on the Bray–Curtis index. Color group communities sampled from a same inoculation treatment. The intensity of the color is proportional to the time elapsed sinceincubation started (8 days, 19 days, 28 days and 40 days, the darker the older).

Bacterial community composition in the wood changed with time of incubation under all three experimental conditions (Fig. 2). In addition, the communities that developed in the different experimental conditions were different from each other at all of the sampling times (multiple PERMANOVA, p < 0.05). However, at the start of the experiment, the wood communities were similar to each other (Supplementary Fig. 1), then, after 19 and 28 days, they mainly became aligned according to the experimental conditions rather than the time of incubation (Supplementary Fig. 1). At the end of the experiment, the bacterial communities of the logs from the open circulation aquarium displayed the greatest difference from the others. At 40 days, the communities from the inoculum at 3 m and 500 m were the most similar to each other (Fig. 2, Supplementary Fig. 1). The communities became more widely dispersed over time for the 500 m inoculum and the open circulation aquarium (Supplementary Fig. 2). For the 3 m inoculum, the dispersion of the communities remained constant over time.

**Figure 3:**
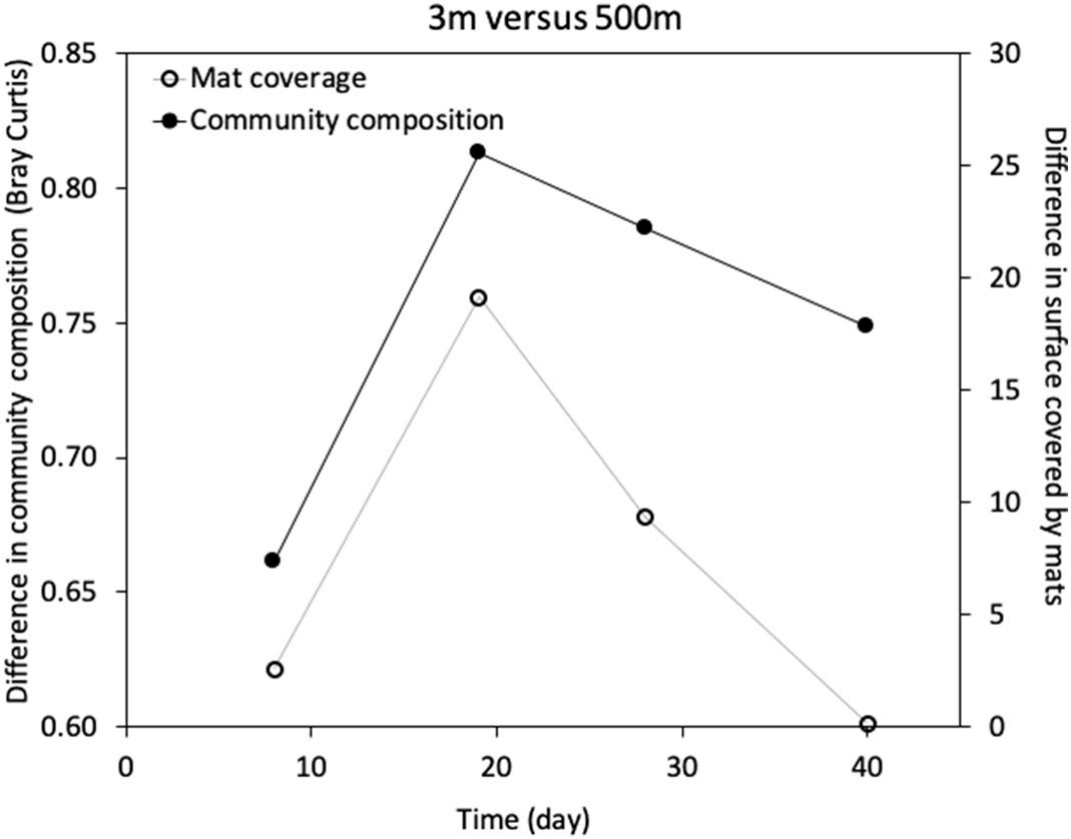
Difference in bacterial community composition based on Bray Curtis dissimilarity compared to the difference in mat coverage between 3 and 500 m depth inoculum experiments.

We then compared the similarity of the composition of the communities in the wood and on the wood surface covered by the mats. The comparison showed that the 3 m and 500 m inoculum wood communities were more similar to each other at the beginning of the incubation when there was almost no difference in the area covered by the mats (Fig. 3). The difference in community composition correlated with the difference in mat coverage after 19 days of incubation, and the difference in community composition was lower again when the difference in mat coverage was lower at the end of the experiment (all logs where almost fully covered by mats) (Fig. 3).

**Figure 4:**
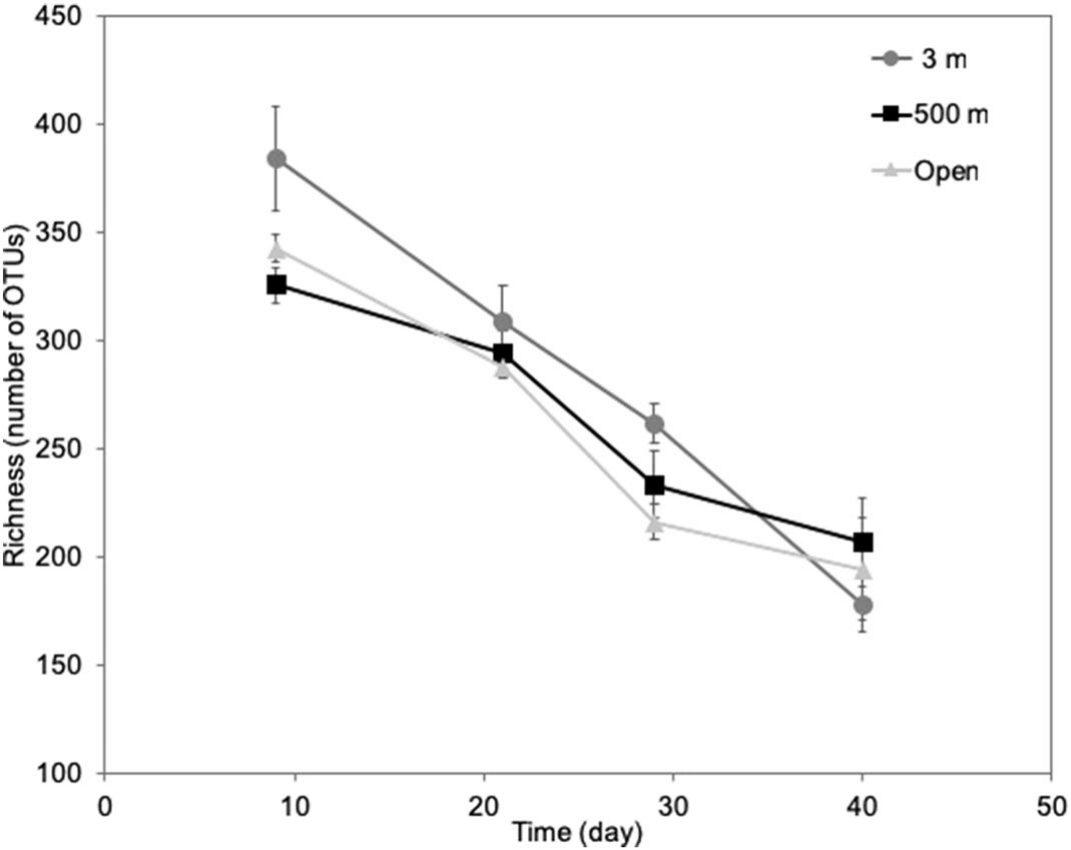
Richness of bacterial communities in the wood for the different experimental conditions and at different times of incubation.

The richness of the bacterial communities in the wood decreased significantly over time in all three experimental conditions (ANOVA, p < 0.05) (Fig. 4), but community richness did not differ between the different experimental conditions.

**Figure 5:**
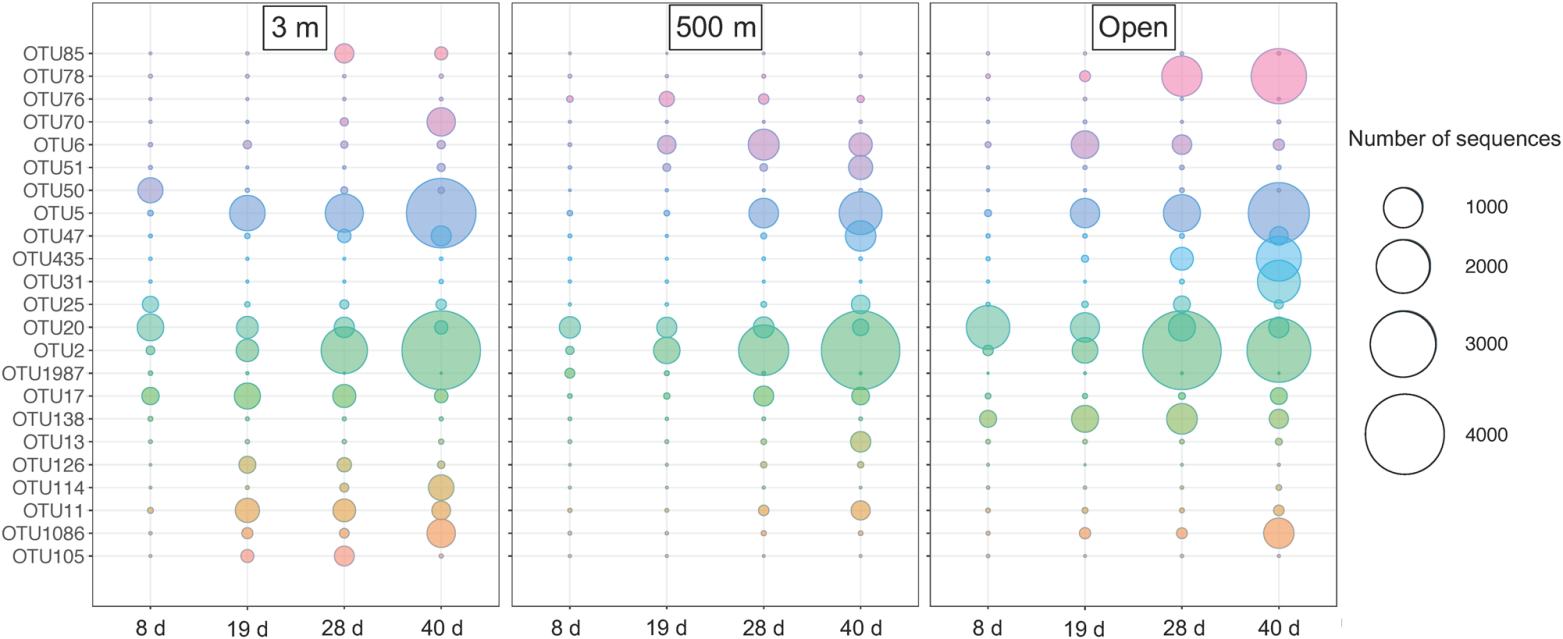
Relative proportion of the main bacterial OTUs in the wood at different times of incubation in aquaria inoculated with water from 3 m depth, 500 m depth or in open circulation.

Using SIMPER statistics, we identified the abundant OTUs (> 1%) that contributed most to differences in bacterial community composition between experimental conditions. Among the most abundant OTUs, the relative proportion of several of them increased over the course of the experiment (Fig. 5). In particular, this was the case for OTU5 identified as *Desulfovibrio* sp (Supplementary Table 1) and OTU2 identified as *Marinifilum* or OTU47 (100% similarity with *Desulfovibrio piezophilus*). These 2 OTUs appeared later in the 500 m inoculum (Fig. 5). Other OTUs, like OTU20 (*Vibrionaceae*, Supplementary Table 1), decreased in abundance over time. Some OTUs were specific for some experimental conditions: OTU85 (*Rhodospirillaceae*) and OTU105 (*Leptotrichiaceae*) were more abundant at 3 m, OTU76 (*Arcobacter*) and OTU51 (*Arcobacter*) in 500 m inoculum, and OTU78 (*Clostridiales*) in the open circulation aquarium (Fig. 5).

### Microbial community potential functions

**Figure 6:**
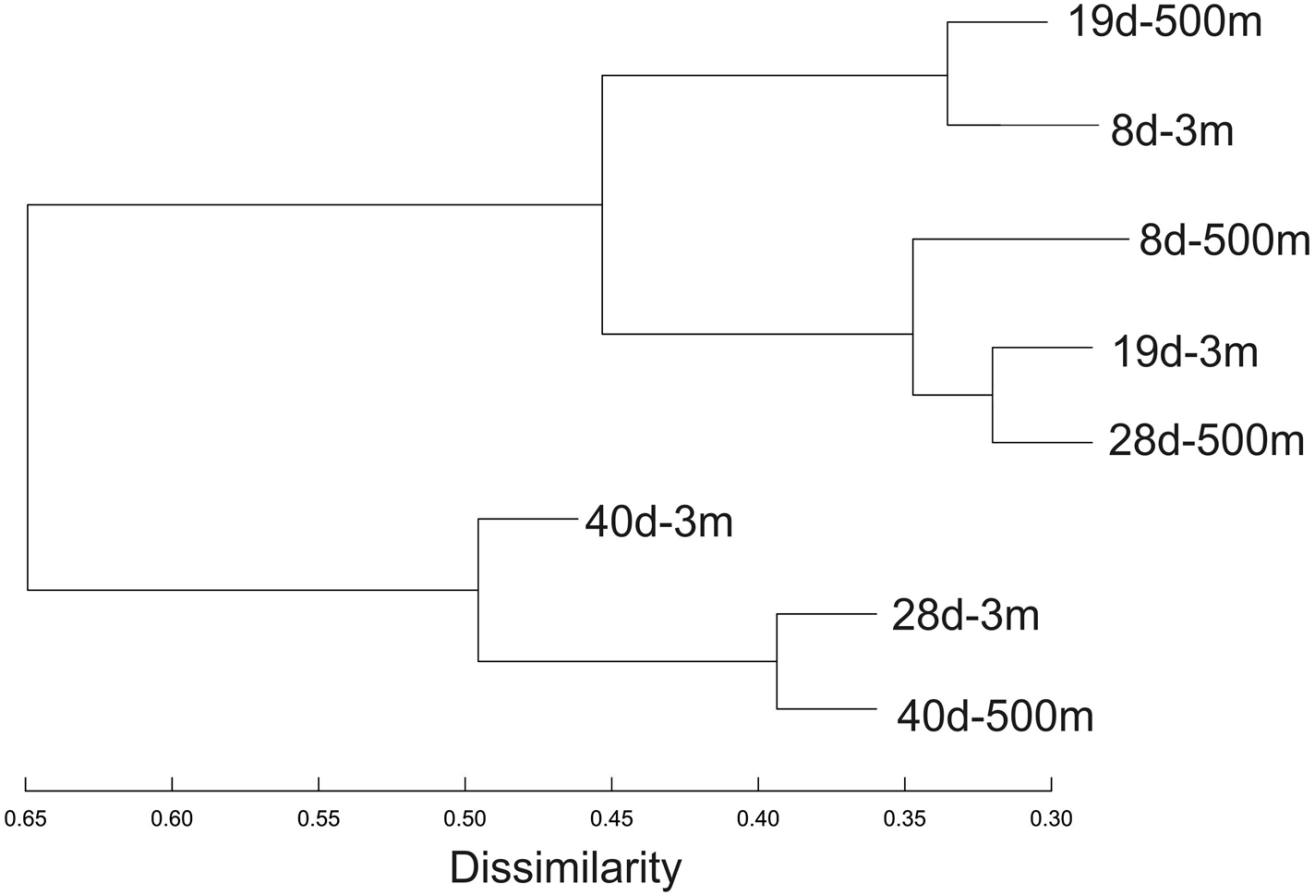
Dendrogram representing the Bray–Curtis similarity between metagenomes obtained from communities sampled after 8 days (8d), 19 days (19d), 28 days (28d) and 40 days (40d) from aquaria inoculated with water from 3 or 500 m depth.

One metagenome was sequenced for each sampling interval for the 3 m and 500 m closed inoculum experiment. We compared the similarity of these metagenomes based on direct comparisons at the k-mer level (Fig. 6). The composition of the metagenomes changed over time for inoculum at both the 3 m and 500 m depth. The dendrogram isolated two main clusters: one containing all samples taken after 8 and 19 days and the other containing samples from the 3 m inoculum taken after 28 and 40 days (28d-3 m and 40d-3 m) along with the sample from the 500 m inoculum taken after 40 days (40d-500 m). The 500 m depth metagenome composition lagged behind the 3 m depth inoculum: 19d-500 m grouped with 8d-3 m, 28d-500 m grouped together with 19d-3 m and 40d-500 m grouped with 28d-3 m (Fig. 6).

**Figure 7:**
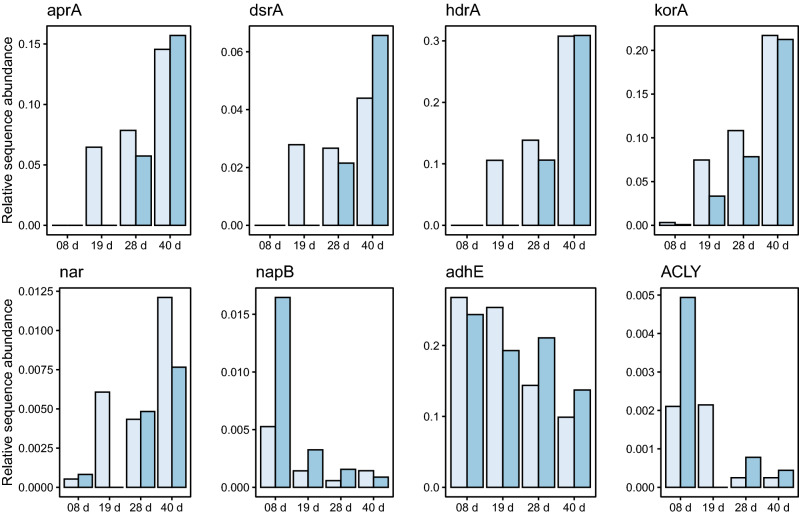
Relative abundance of selected KEGG annotated functional genes within wood samples at different times of incubation in aquaria inoculated with water from 3 m depth (light blue) and 500 m depth (dark blue).

We also identified genes involved in pathways that we had identified earlier as being associated with wood degradation in marine ecosystems^25^. In the 3 m and 500 m closed inoculum experiments, some genes increased in relative abundance over time: *aprA* (adenylylsulfate reductase), *dsrA* (dissimilatory sulfite reductase), *hdrA* (heterodisulfide reductase), *korA* (ketoglutarate ferredoxin oxidoreductase) and *nar* (respiratory nitrate reductase) (Fig. 7). These genes were first detected after 19 days in the 3 m inoculum. They were seen later and at a relatively lower abundance in the 500 m inoculum. After 40 days, the relative abundance of these genes was similar or higher in the 500 m inoculum as in the 3 m inoculum (Fig. 7).

Another group of genes decreased in relative abundance over time: *napB* (periplasmic nitrate reductase), *adhE* (acetaldehyde/alcohol dehydrogenase) and *ACLY* (ATP citrate lyase). *napB,* the latter of which was relatively more abundant in the 500 m inoculum. *adhE* was relatively more abundant in the 3 m inoculum at the beginning of the incubation and then became more abundant in the 500 m inoculum after 28 days (Fig. 7).

## Discussion

Our experiment whose objective was to test the effect of historical contingencies on marine microbial assemblies, showed that history, or the legacy effect, significantly influenced microbial community assembly. The same substrate, wood, immersed in sea water, was colonized by bacteria with different evolutionary and ecological histories and resulted in communities of varying composition. The 3 m and 500 m inoculum communities represent assemblages of bacteria that had been historically separated, and whose historical legacies had been maintained because of the environmental dissimilarity between surface and deep sea waters. We demonstrate that historical contingencies influence the community composition over time, as well as influencing the microbial activity, measured by growing mat area, and the potential metabolisms of the communities.

A long-standing question in ecology is whether a strong one-to-one match is found between environment and community^6^. In other words, it raises the question of whether two different sets of species that colonize localities with similar environmental conditions will result in similar community composition. In our experiment, different inoculum communities result in different compositions of microbial communities in the wood. However, the differences observed between the wood assemblies were greater at the beginning of the experiment than at the end. Thus, the timeframe and magnitude of the response appear to be powerful components of the historical contingency effect on community assembly. A detailed observation of these differences showed that the microbes originating from 500 m depth had a lagged functional response compared to the microbes originating from 3 m depth. At the end of the experiment, however, the similarity between the activity and functional characteristics appeared greater for all communities, even though community composition still differed. It indicates that the effect of historical contingencies was mostly transient in the case of microbes colonizing wood. Because of wood’s specific chemical and physical composition, microbial growth on wood is constrained during both terrestrial^43^ and marine degradation^31,44^. Similarities between communities increased after 20 days in our experiment. At the OTU level, both treatments then hosted the same dominant taxa, which indicates that the effect of environmental selection was so strong that, over time, species sorting became the important driver of community assembly.

In the field of community ecology, studies that empirically evaluate community assembly have demonstrated cases where communities converge to a similar species composition, even when the order in which the species enter a community varies. These earlier studies include highly controlled laboratory experiments on plankton^45^, field experiments with plants^46^ and observations in nature^47,48^. In contrast, many other studies have shown that even under similar environmental conditions, very different communities can develop under controlled laboratory experiments, in field experiments and in nature^50–52^ as a result of variation in the origin, timing and sequence of species colonization^49^. Thus, in some cases, it is believed that communities converge to form a unique assembly, unrelated to immigration history, whereas in other cases, communities achieve multiple stable assemblies. There are fewer studies on microorganisms but results have recently been reviewed to define the processes that can produce legacy effects^19^, including dispersal, historical contingency and the timing of colonization. Our results add to the growing body of evidence that historical contingency have an effect on community assembly. In addition, our study takes a step further by gathering data from a marine ecosystem where the effect of historical contingencies is shown to be transient.

The microorganisms that colonize wood in seawater are considered to be ultra-rare members of the planktonic microbial community^26^. They probably remain inactive or dormant in seawater until they encounter a favorable substrate that they can colonize. One hypothesis to explain the differences between the various inoculum observed in our experiment could be that the number of potential colonizers is lower at 500 m depth than at 3 m depth. The shallow coastal areas receive regular terrestrial inputs, including wood, brought by rivers. It could explain the presence of a larger pool of specialized organisms in shallow coastal water compared to the deep sea. The reduced prevalence of these ultra-rare microbes in the deep would reduce the potential number of organisms available to colonize the wood. This would in turn slow down the development of the specialized wood microbe community and reduce the resulting sulfur-related ecosystem functions. The organisms in shallow water may also be “fresher” and thus more active than the possibly dormant organisms found in the deep sea. The dormant species may have needed more time to revive, grow and become active in the wood community.

In contrast to the differences observed in community composition, we did not detect differences in community diversity between treatments. All showed decreasing diversity as the time of incubation increased as reported earlier in the wood experiments^26^. This could reflect a reduction in the number of microbial niches concomitant with the development of a community of specialized organisms adapted to this particular habitat.

Another objective of our experiment was to test the specific consequences of the priority effect on community assembly. The underlying hypothesis is that the history of the colonizers is not the only factor that matters, but that the order and timing of the arrival of the colonizers may also impact on the final community assembly. Our experiment showed that in the 3 m open circulation experiment which promotes the successive arrival of different communities over time, the mats started growing later than in the 3 m closed experiment. It suggests that the priority effect has an impact on the activity and function of the microbial assemblies. One explanation for the difference observed between the closed inoculum and the open circulation experiment may be that, by regularly allowing new microbes to colonize the wood, different patterns of competition or mutualism can take place that could change the overall succession of microorganisms in the wood and the subsequent degradation process. In addition, the chance of encounter between ultra-rare microbes and the wood is reduced when water is renewed constantly. The rare microbes may need a higher residence time to increase the chance of encounter with the substrate. The importance of the priority effect for microorganisms has been shown in terrestrial ecosystems^53–55^, but studies on natural communities and in aquatic ecosystems are rare. Experimental studies in freshwater have shown that the history of community assembly affects the productivity-biodiversity relationship^56^, and that the effect can be variable and not necessarily predictable^21^. Other studies have shown the importance of the timing of dispersal^20^ or have focused specifically on selection based on past environmental conditions^17,18^, which could be a result of the priority effect^22^. Our study, to the best of our knowledge, is the first to report on the effect of historical contingencies, and more specifically the priority effect, on marine microbial communities. Future studies, including more replicates, could help to elucidate the precise effect that historical contingencies have on the kinetics of the microbes responsible for rapid chemical and physical changes in the marine environment.

We observed the same key OTUs in both 500 m and 3 m, in particular, in emblematic microorganisms of chemosynthetic ecosystems, such as the sulfate-reducing *Desulfovibrio piezophilus* originally isolated from wood falls^57^. It suggests that these specialized microorganisms, although ultra-rare in the sea, are distributed globally by different water masses. The dynamics of the OTUs found in both 500 m and 3 m inoculum differed between experiments, however, which may be due to the factors discussed above. Interestingly, some OTUs were specific to some inoculum. Within *Arcobacter* for instance, known to be potential sulfur-oxidizers^25,58^, different OTUs were seen in the inoculum from 3 m depth and from 500 m depth. This indicates that while historical contingency promotes different bacterial species, it has the same potential to oxidize sulfur. These species that are able to colonize wood are probably not functionally redundant even though they have some common metabolisms^4^. The fact that the activity (growth of mats) and the dynamics of specific potential metabolisms differed may be due to the presence of less-efficient microorganisms in the 500 m depth inoculum.

The microbes colonizing the wood comprise a succession of metabolisms reflecting different steps in the degradation of the wood^31^. We detected genes associated with anaerobic fermenters that take advantage of the sugars present in the sap, and syntrophic bacteria that cross-feed on waste products from fermenters. We also detected methanogens that use products of fermentation for growth and respiration, ultimately producing methane. The other key species that we detected were associated with the sulfur cycle. These were sulfate reducers that respire the sulfate present in seawater and sulfur oxidizers that can oxidize the hydrogen sulfur produced in the wood when they are in contact with the oxygen found in sea water. The metagenomic approach allowed the diagnostic genes for all these metabolisms to be detected and their dynamics to be assessed between experiments. It highlights for the first time an effect of historical contingency, not only on the community composition, but also on the functional dynamics of the communities in an essential transient deep-sea ecosystem.

## Supplementary Information

Supplementary Figures.

Supplementary Table 1.
