# Supplementary material for: Historical contingency impacts on community assembly and ecosystem function in chemosynthetic marine ecosystems
Source: Sci Rep. 2021 Jul 7;11:13994. doi: 10.1038/s41598-021-92613-1 (PMC8263718; doi:10.1038/s41598-021-92613-1)
Supplement: Supplementary file 1 — Supplementary Figures. [file 41598_2021_92613_MOESM1_ESM.docx]

Historical contingency impacts community assembly and ecosystem function in chemosynthetic marine ecosystems

Dimitri Kalenitchenko^1^, Erwan Peru^2^, Pierre E Galand^2^

^1^Centre for Arctic Gas Hydrate, Environment and Climate, UiT – The Arctic University of Norway, Department of Geosciences, Tromsø, Norway

^2^Sorbonne Université, CNRS, Laboratoire d’Ecogéochimie des Environnements Benthiques, LECOB, 66500 Banyuls-sur-Mer, France.

^*^ Correspondence: Pierre E Galand, [pierre.galand@obs-banyuls.fr](mailto:pierre.galand@obs-banyuls.fr); Dimitri Kalenitchenko, [dimitri.kalenitchenko@gmail.com](mailto:dimitri.kalenitchenko@gmail.com)

Supplementary Fig. 1. Dendrogram showing the similarity between wood bacterial community composition at T1 (8 days), T2 (19 days), T3 (28 days) and T4 (40 days) under the 3 different experimental conditions: 3 m inoculum (3 m), 500 m inoculum (500 m) and open circulation inoculum (Open). Numbers at the end of the names indicate replicates.

Supplementary Fig. 2: Boxplots representing the betadispersion of the bacterial communities under each of the 3 experimental conditions at T1 (8 days), T2 (19 days), T3 (28 days) and T4 (40 days). The bar represent the median distance to centroid and the boxes represent upper and lower 25% quartiles. The whiskers represent minimum and maximum values.
